# Sphingosine-1-Phosphate-Specific G Protein-Coupled Receptors as Novel Therapeutic Targets for Atherosclerosis

**DOI:** 10.3390/ph4010117

**Published:** 2011-01-04

**Authors:** Yasuo Okamoto, Fei Wang, Kazuaki Yoshioka, Noriko Takuwa, Yoh Takuwa

**Affiliations:** Department of Physiology, Kanazawa University Graduate School of Medical Science, 13-1 Takara-machi, Kanazawa, Ishikawa 920-8640, Japan; E-Mails: wf_0908@hotmail.com (F.W.); yoshioka@med.kanazawa-u.ac.jp (K.Y.); ntakuwa@ishikawa-nu.ac.jp (N.T.)

**Keywords:** sphingosine-1-phosphate, S1P_1_, S1P_2_, S1P_3_, atherosclerosis, macrophages, endothelial cells, lymphocytes

## Abstract

Atherosclerosis is a chronic inflammatory process involving complex interactions of modified lipoproteins, monocyte-derived macrophages or foam cells, lymphocytes, endothelial cells (ECs), and vascular smooth muscle cells. Sphingosine-1-phosphate (S1P), a biologically active blood-borne lipid mediator, exerts pleiotropic effects such as cell proliferation, migration and cell-cell adhesion in a variety of cell types via five members of S1P-specific high-affinity G protein-coupled receptors (S1P_1_-S1P_5_). Among them, S1P_1_, S1P_2_ and S1P_3_ are major receptor subtypes which are widely expressed in various tissues. Available evidence suggest that S1P and HDL-bound S1P exert atheroprotective effects including inhibition of leukocyte adhesion and stimulation of endothelial nitric oxide synthase (eNOS) in endothelial cells (ECs) through the activation of G_i_ signaling pathway via S1P_3_ and probably S1P_1_, although there is still controversy. FTY720, the phosphorylation product of which is a high-affinity agonist for all S1P receptors except S1P_2_ and act as an immunosuppressant by downregulating S1P_1_ on lymphocytes, inhibits atherosclerosis in LDL receptor-null mice and apoE-null mice through the inhibition of lymphocyte and macrophage functions and probably stimulation of EC functions, without influencing plasma lipid concentrations. In contrast to S1P_1_ and S1P_3_, S1P_2_ facilitates atherosclerosis by activating G_12/13_-Rho-Rho kinase (ROCK) in apoE-null mice. S1P_2_ mediates transmigration of monocytes into the arterial intima, oxidized LDL accumulation and cytokine secretion in monocyte-derived macrophages, and eNOS inhibition and cytokine secretion in ECs through Rac inhibition, NF-κB activation and 3′-specific phosphoinositide phosphatase (PTEN) stimulation downstream of G_12/13_-Rho-ROCK. Systemic long-term administration of a selective S1P_2_-blocker remarkably inhibits atherosclerosis without overt toxicity. Thus, multiple S1P receptors positively and negatively regulate atherosclerosis through multitudes of mechanisms. Considering the essential and multi-faceted role of S1P_2_ in atherogenesis and the impact of S1P_2_ inactivation on atherosclerosis, S1P_2_ is a particularly promising therapeutic target for atherosclerosis.

## 1. Introduction

It is increasingly recognized that atherosclerosis is a complex chronic inflammatory disease rather than a mere phenomenon of lipid deposition on the vascular wall [1,2]. In endothelial cells (ECs), proinflammatory stimuli, including hypercholesterolemia, hyperglycemia and smoking, trigger the expression of adhesion molecules such as vascular cell adhesion molecule-1 (VCAM-1) and selectins, which mediate the attachment of circulating monocytes and lymphocytes to ECs. Chemokines and other cytokines, which are produced by vascular wall cells, elicit the infiltration of adherent leukocytes into the intima. Within the intima, monocytes differentiate into macrophages and engulf modified low density-lipoprotein (LDL) through scavenger receptor-mediated endocytosis, leading to conversion of macrophages into lipid-laden macrophages, foam cells. Macrophages amplify the inflammatory responses through the release of numerous cytokines and growth factors. T cells also enter lesions and amplify the local inflammatory responses by producing proinflammatory cytokines. The cytokines and growth factors secreted by these leukocytes as well as ECs direct migration of vascular smooth muscle cells (SMCs) into lesions. In the intimal lesions, SMCs proliferate under the influence of various growth factors and release collagens and other extracellular matrices, expanding the lesions. In advanced atherosclerotic lesions, increased inflammatory activities diminish the collagen content and increase procoagulant activity, leading to plaque rupture and acute coronary thrombosis.

Sphingosine-1-phosphate (S1P) is a blood borne, lysophospholipid mediator that exerts pleiotropic activities including cell proliferation, survival, migration, cell shape and cell-cell adhesion in a variety of cell types [3-6,7]. S1P was originally shown to be released from activated platelets [8] and to be present in the plasma at around 10^−7^∼10^−6^ mol/L, largely in a form bound to plasma proteins, albumin and high density-lipoprotein (HDL) [8-10]. In agreement with this, plasma S1P levels were highly correlated with HDL concentrations [10]. A number of investigations provided evidence for the notion that HDL possesses an atheroprotective activity [11-13]. It is established that HDL plays an essential role in cholesterol efflux from cholesterol-laden macrophages as a cholesterol acceptor and cholesterol transport to the liver [13]. Besides this role of HDL, it is suggested to exert atheroprotective effects through the HDL-bound S1P [14-16]. Previous studies showed that HDL-bound S1P and other sphingolipids mediated the atheroprotective actions of HDL, including EC survival and proliferation, stimulation of endothelial nitric oxide (NO) synthase (eNOS), inhibition of endothelial expression of VCAM-1 and intracellular adhesion molecule (ICAM)-1, and inhibition of monocyte chemoattractant peptide (MCP)-1 production in SMCs [14-16]. In contrast, other studies showed that S1P exhibits proatherogenic activities. For example, the proinflammatory cytokine tumor necrosis factor-α (TNF-α) activated EC, as evaluated by the expression of adhesion molecules such as E-selectin and VCAM-1, through a sphingosine kinase (SphK), which is an S1P-synthezing enzyme (see below for details) [17]. HDL inhibited the expression of these adhesion molecules by suppressing SphK [18].

Although many reports have suggested that S1P may be involved in atherosclerosis, it remained undefined whether S1P is proatherogenic or antiatherogenic and by what mechanisms S1P modifies atherosclerosis [19]. Recent studies including ours have addressed the *in vivo* roles of S1P receptor subtypes in atherosclerosis using mouse models of atherosclerosis [20-23]. In this review, we will focus on the receptor subtype-specific, stimulatory and inhibitory effects of S1P on atherosclerosis and discuss the possibility of using S1P receptors as a novel therapeutic target for atherosclerosis.

## 2. S1P Metabolism, Receptors and Their Actions

### 2.1. Synthesis and Degradation of S1P

S1P is generated from sphingomyelin, an integral component of plasma membranes, by the sequential action of sphingomyelinase, ceramidase, and sphingosine kinases [24]. The SphK1 and SphK2, rate-limiting enzymes for S1P synthesis, catalyze the phosphorylation of sphingosine to produce S1P [25,26]. The SphK1 and SphK2 exhibit different expression patterns and kinetic properties and may therefore regulate different S1P-dependent processes. SphK1/SphK2 double knockout mice are embryonic lethal and virtually lack tissue S1P, indicating that S1P is produced exclusively by SphKs [27,28]. Degradation of S1P occurs by the dephosphorylation by S1P phosphatase (SPP) and the cleavage to palmitoaldehyde and phosphoethanolamine by S1P lyase [29,30]. Once synthesized, S1P is released from cells through the export across the cell membrane likely via the ATP-binding cassette (ABC) family of transporters such as ABCC1, ABCA1 and ABCG2 [31-33] and recently identified “protein two of hearts” (also known as spns2) [34,35].

The major constitutive source of plasma S1P is red blood cells with additional contribution of non-hematopoietic cells including vascular endothelial cells [36-39]. Lymphatic ECs and neural crest-derived pericytes in the thymus are constitutive sources for S1P in lymph and the thymic local milieu, respectively [40,41], while activated platelets, mast cells, macrophages and other cell types were reported to produce and secrete S1P upon stimulation [8,42,43]. S1P in plasma and locally produced by macrophages, mast cells, ECs, and other cells in lesions could be involved in atherosclerosis.

### 2.2. S1P Receptors and Their Actions

**Figure 1:**
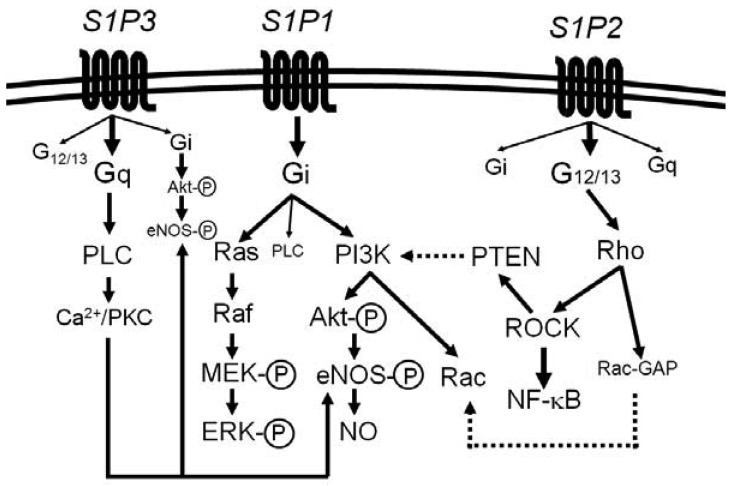
S1P receptor signaling. S1P_1_ couples exclusively to G_i_, whereas S1P_2_ and S1P_3_ couple to multiple G proteins. The G proteins preferred by S1P_2_ and S1P_3_ are G_12/13_ and G_q_, respectively. G_i_ couples to stimulation of phosphoinositide 3-kinase (PI3K)-Akt/Rac pathway and Ras-mitogen activated protein kinase pathway, G_12/13_ couples to activation of Rho pathway, mediating Rac inhibition, NF-κB activation and PTEN stimulation. G_q_ mediates stimulation of phospholipase C (PLCβ) pathway. Nitric oxide synthase (eNOS) in ECs is fully activated by G_i_- and Akt-mediated phosphorylation of eNOS in concert with a G_q_-mediated, Ca^2+^/calmodulin-dependent activation. The straight and dotted lines show the stimulatory and inhibitory regulations, respectively.

Most of the diverse biological activities of S1P are mediated by five members of S1P-specific high-affinity G protein-coupled receptors, S1P_1_ [or S1PR1 (gene name)] − S1P_5_ (or S1PR5) [44-48]. S1P_1_, S1P_2_ and S1P_3_ are widely expressed in various tissues and the major receptor subtypes in the vasculature [6,7,22,44,45]. The diversity of responses to S1P depends on their subtype-specific, differential coupling to various G-proteins, in combination with tissue- and cell type-specific receptor expression patterns (Figure 1) [44-46,49-52].

S1P_1_ couples exclusively to G_i_, whereas S1P_2_ and S1P_3_ couple to multiple G proteins but the G proteins preferred by S1P_2_ and S1P_3_ are G_12/13_ and G_q_, respectively [49-55]. Downstream of the heterotrimeric proteins, S1P_1_ activates phosphoinositide 3-kinase (PI3K)-Akt/Rac pathway and Ras-mitogen activated protein kinase pathway, S1P_2_ activates Rho-Rho kinase (ROCK) pathways including NF-κB and the 3′-specific phosphoinositide phosphatase, phosphatase and tensin homolog (PTEN), and S1P_3_ activates phospholipase C (PLC) pathway [49-55]. Moreover, intracellular S1P may regulate cell growth, survival and other functions in a receptor-independent manner [43,56,57]. It is unknown whether and how such intracellular mechanisms of S1P contribute to vascular physiology and diseases.

### 2.3. S1P Receptors in ECs

ECs, SMCs, lymphocytes and monocytes/macrophages, which are involved in atherogenesis, show distinct patterns of the expression of S1P_1_, S1P_2_ and S1P_3_. ECs express easily detectable levels of S1P_1_ and S1P_3_, whereas S1P_2_ expression appears to be relatively low [58,59]. ECs release the atheroprotective mediator NO [60]. S1P activates eNOS likely via S1P_3_ and probably S1P_1_ to stimulate NO production [61,62]. S1P also maintains endothelial barrier function via S1P_1_ through the facilitating effect on adherens junctional assembly [63-66].

S1P was reported to positively and negatively regulate the monocyte-EC interaction through multiple mechanisms. S1P stimulated the expression of the adhesion molecules VCAM-1 and ICAM-1 in human umbilical vein ECs (HUVECs) and monocyte adhesion in G_i_- and NF-κB-dependent manner [15,67]. The down-regulation of S1P_1_ signaling by siRNA knockdown decreased the induction of E-selectin after tumor necrosis factor-α (TNF-α) or lipopolysaccharide (LPS) stimulation of human ECs [68]. In contrast, it was reported that S1P inhibited EC activation; S1P-containing HDL inhibited the induction of endothelial adhesion molecules by TNF-α through NO pathway in HUVECs [14,15]. In agreement with an inhibitory effect of S1P on adhesion molecules expression in ECs, S1P_1_ agonist SEW2871 suppressed the adherence of inflammatory mononuclear cells to TNF-α-activated aortic ECs [69,70]. In diabetic NOD mice, S1P and SEW2871 activated S1P_1_ to abrogate monocyte adhesion to aortic ECs in a partially NO-dependent manner and VCAM-1 expression due to their inhibitory effect on NF-κB [71]. S1P also suppressed the adhesion of monocytic cell line U937 to HUVECs via the endothelial integrins α5β1 and αvβ3, independently from the expression of adhesion molecules [72]. In addition, S1P stimulated the expression of interleukin (IL)-8 and MCP-1, chemoattractants for leukocytes, via S1P_1_ and S1P_3_ in HUVECs [73,74].

These results suggest that when the cells are exposed to exogenous S1P, the expression level of adhesion molecules and chemokines may be determined by both the NF-κB-mediated stimulatory signal and the NO-mediated inhibitory signal. The net effects of S1P on leukocyte adhesion to ECs, chemokine production, and consequent leukocyte infiltration into the intima may be affected by differences in S1P receptor expression.

### 2.4. S1P Receptors in SMCs

In atherosclerosis, SMCs are involved in plaque expansion and its stabilization by migrating to form a fibrous cap over the plaque and preventing it from rupture [1,2]. SMCs from adult vessels express S1P_2_ and S1P_3_, while SMCs from pups express S1P_1_, S1P_2_, and S1P_3_ [58,75]. S1P_1_ mediates migration and proliferation in response to S1P [75,76]. Our previous observations demonstrated that S1P inhibited platelet-derived growth factor (PDGF)-induced migration in adult SMCs through S1P_2_-G_12/13_-Rho-dependent Rac inhibition [52,54,77]. In agreement with inhibitory effect of S1P_2_ on migration of SMCs, the enhanced neointimal lesion formation was induced by ligation of the carotid artery in S1P_2_^−/−^ mice, and higher rate of *in vitro* proliferation and migration in S1P_2_^−/−^ SMCs was observed [78]. S1P_2_ also promoted the SMC differentiation, thereby limiting the growth potential of SMCs [79,80]. Activated SMCs are an abundant source of proatherogenic cytokines and chemokines including MCP-1 [1,2]. S1P and S1P-containing HDL inhibited NAD(P)H oxidase-dependent reactive oxygen species generation and MCP-1 production via S1P_3_ in SMCs [16,81].

### 2.5. S1P Receptors in Monocytes/Macrophages

Circulating monocytes and monocyte-derived macrophages play a crucial role in atherosclerosis by adhering to activated ECs, transmigrating into the intima, and differentiating into macrophages and lipid-laden foam cells [1,2,82]. Monocytes and macrophages express multiple S1P receptors [83-86], but show species-specific difference. Human monocytes express S1P_1_, S1P_2_ and S1P_4_, and human macrophages express S1P_1_-S1P_4_ [83], while murine bone marrow (BM)-derived macrophages mainly express S1P_1_ and S1P_2_ [22,84-86]. S1P_2_ mediates inhibition of C5a-induced migration of murine primary macrophages *in vitro*, and macrophages isolated from S1P_2_-knockout (S1P_2_^−/−^) mice displayed enhanced recruitment during thioglycollate-induced peritonitis [87]. S1P may positively and negatively regulate the transmigration of monocytes and macrophages into the intima via S1P_1_ and S1P_2_.

Recent studies indicated that SphK activation is involved in inflammatory responses via the action of the intracellular S1P in macrophages [56,57], whereas extracellular S1P predominantly triggers anti-inflammatory responses via binding to cell surface S1P receptors [88]. S1P selectively attenuates Toll-like receptor 2 signaling via S1P_1/2_-mediated negative cross-talk in murine macrophages, thus preventing macrophage activation [89]. S1P also promoted the conversion of macrophages from the proinflammatory (M1) to anti-inflammatory (M2) phenotype with S1P_1_-mediated inhibition of LPS-induced secretion of TNF-α, MCP-1 and IL-12 in murine peritoneal macrophages [86]. These results suggest that S1P facilitates the anti-inflammatory signal generation in macrophages via S1P_1_.

Oxidized LDL exerts cytotoxic effects to induce apoptosis in macrophages [90]. The environment within atherosclerotic lesions is extremely proapoptotic. As for other cells, S1P protects macrophages against apoptosis [91,92]. S1P, which is derived from apoptotic cells, activates PI3K, ERK and Ca^2+^ signaling in macrophages to protect them against apoptosis through the heme oxygenase 1-dependent upregulation of the anti-apoptotic proteins Bcl-2 and Bcl-xL [91,92].

### 2.6. S1P Receptors in Lymphocytes

The circulation of mature lymphocytes between blood and secondary lymphoid tissues is a central process in the immune surveillance. The immunosuppressant FTY720 (fingolimod), a structural analogue of sphingosine, which is phosphorylated *in vivo* by SphK2 [93]. The phosphorylated product of FTY720 (FTY720-P) is a high-affinity agonist for S1P_1_, S1P_3_, S1P_4_ and S1P_5_ but not S1P_2_, depletes lymphocyte from the blood by binding to and downregulating S1P_1_ on both T and B lymphocytes [38,94-96]. FTY720 is called a functional antagonist after this action. FTY720 has recently been officially approved as an orally available therapeutics for multiple sclerosis in U.S.A. and Russia [97].

## 3. Effects of FTY720 on Atherosclerosis

FTY720-P acts on S1P_1_ and induces immunosuppression by sequestering lymphocytes, particularly T lymphocytes, in secondary lymphoid organs and decreasing circulating lymphocytes. T lymphocytes are involved in the initiation and progression of atherosclerosis [1,2]. EC-derived NO is an atheroprotective mediator [60] and upregulated by endothelial S1P_3_ and probably S1P_1_ [61,62]. Therefore, it is rational to hypothesize that FTY720 may have an impact on atherosclerosis through its immunosuppressing and eNOS-stimulating effects.

Two groups tested the pharmacological actions of FTY720 on the initiation and progression of atherosclerosis in atherosclerotic models, apoE^−/−^ mice and LDLR^−/−^ mice [20,21]. Keul *et al.* demonstrated that oral FTY720 administration (1.25 mg/kg body weight/day) for 20 weeks resulted in more than a 50% reduction of atherosclerotic lesion volumes in apoE^−/−^ mice fed a high fat (Western) diet without any influence on plasma lipid concentrations [21]. The reduction of atherosclerotic lesions was accompanied by decreases in macrophage density and collagen deposition in the lesions but not changes in the density of CD3^+^ T lymphocytes or SMCs. As expected, FTY720 administration induced lymphopenia, indicating that FTY720 induced immunosuppression by effectively downregulating lymphocyte S1P_1_. In contrast, S1P_3_-mediated, NO-dependent vasodilator response of aortae isolated from FTY720-administered mice to acute FTY720 challenge remained intact, suggesting that chronic FTY720 administration did not downregulate endothelial S1P_3_ or compromise S1P_3_-mediated eNOS activation. In addition, they showed that FTY720-P treatment of isolated aortic segments and cultured SMCs potently inhibited thrombin-induced MCP-1 release. Thrombin-induced MCP-1 response was abolished in tissues and cells from S1P_3_-null mice. The gene expression of cytokines including IL-12, IL-10, IL-4 or IFN-γ in isolated peritoneal macrophages was not different between mice receiving FTY720 and vehicle. Based on these observations, they suggested that FTY720 inhibited atherosclerosis mainly by suppressing monocyte/macrophage recruitment to atherosclerotic lesions through mechanisms involving S1P_3_-mediated, probably NO-dependent inhibition of MCP-1 production.

Nofer *et al.* has demonstrated in high cholesterol diet (HCD)-fed LDLR^−/−^ mice that intraperitoneal injection of FTY720 three times a week (0.04 or 0.4 mg/kg body weight/day) for 16 weeks reduced atherosclerotic lesions in a dose-dependent manner [20]. FTY720 substantially decreased CD3^+^ T lymphocytes in lesions but not affect macrophage density, smooth muscle density or collagen content. Moreover, FTY720 inhibited necrotic core formation. FTY720 only at the high dose lowered the peripheral blood lymphocyte with a preferential decrease in T cells, particularly CD4^+^ helper T cell subset. The plasma level of the T cell cytokine IFN-γ was reduced with diminished concanavarin A-induced *in vitro* mitogenesis of lymphocytes from mice receiving either the low or high dose of FTY720, suggesting that FTY720 attenuated Th1 immune responses. Analogous to lymphocytes, the plasma levels of the macrophage-derived cytokines, TNF-α, IL-6 and IL12, were reduced in mice receiving FTY720 although macrophage accumulation in plaques was not altered in FTY720-treated mice. These observations collectively suggested that chronic administration of FTY720 attenuates development of atherosclerosis through the inhibition of functions of T cells and macrophages.

Recently, it was shown that in apoE^−/−^ mice on a normal diet, hypercholesterolemia is induced by treatment with a relatively higher dose of FTY720 (3 mg/kg/day) for 12 weeks, which possibly counteracts its anti-atherogenic effect on immune cell distribution [98].

These studies indicate that FTY720 effectively inhibits atherosclerosis, without affecting blood lipid profiles, at the doses which inhibit T cells activity as evaluated with circulating lymphocyte numbers and T cell-specific cytokine production as markers [20,21]. Moreover, the reduced plasma levels of the cytokines, which are abundantly produced by macrophages, suggest that FTY720 directly or indirectly via the regulation of lymphocytes inhibits macrophage activity. In addition, FTY720 may inhibit mobilization of monocytes/macrophages into lesions through mechanisms involving the attenuation of chemokine production. Accumulated evidence indicates that FTY720-induced sequestration of T cells in lymphoid organs and resultant lymphopenia occurs as a result of downregulation of lymphocyte S1P_1_, *i.e.* the functional antagonism of S1P_1_ [38,94-96]. It remains unclear whether chronic administration of FTY720 similarly downregulates macrophage S1P_1_ *in vivo* and consequently induces significant functional changes of macrophages. Besides the immune cells as targets of FTY720, this compound may have non-immune cell targets, including ECs and SMCs, in inhibiting atherosclerosis. FTY720-P activates eNOS to stimulate NO production in ECs via S1P_3_ and probably S1P_1_. In this respect, it is noted that chronic FTY720 administration did not impair FTY720-P-induced vasodilation, which suggest that S1P_3_ and/or S1P_1_ in ECs were not downregulated [21]. It is likely that FTY720-P activates endothelial S1P_3_ and S1P_1_ as a functional agonist, leading to increased release of NO. Likewise, S1P_3_ and S1P_1_ may mediate inhibition of the expression of cytokines including the monocyte chemoattractant MCP-1, leading to inhibition of monocytic infiltration into lesions.

Systemic administration of non-selective immunosuppressive drugs will probably not be useful for the treatment of atherosclerosis because of adverse effects including serious infections [99]. Although FTY720 at a low dose did not reduce circulating lymphocytes [20], it decreased the plasma levels of T cell-specific cytokines, which suggest that FTY720 at the low dose might be accompanied by immune suppression. It is necessary to fully dissect the molecular mechanisms underlying the anti-atherogenic effect of FTY720 at various doses.

## 4. S1P_2_ as a New Target for Treatment of Atherosclerosis

Three major S1P receptors, S1P_1_, S1P_2_ and S1P_3_, are expressed in ECs, SMCs and monocytes/macrophages. Among these, S1P_2_ but not S1P_1_ or S1P_3_ in SMCs and ECs mediate inhibition of chemoattractant-directed cell migration [54,58,100]. This unique functional property of S1P_2_ can be accounted for by the distinct signaling capacity of S1P_2_; differently from S1P_1_ and S1P_3_, which are G_i_-coupled receptors, S1P_2_ couples mainly to G_12/13_ to result in Rho activation, Rho-dependent Rac inhibition and PTEN stimulation, leading to chemorepulsion. Our recent observations [22,101] showed that ECs express a significant level of S1P_2_ *in vivo* although commonly employed HUVECs do not express an easily detectable level of S1P_2_. S1P_1_ and S1P_3_ stimulate eNOS in ECs and inhibit leukocyte adhesion to ECs. In contrast, the Rho- ROCK pathway, which is activated preferentially by S1P_2_, is reported to participate in an inflammatory response [55,102]. In addition, S1P_2_ mediates the opposite effect on migration of monocytes/macrophages to that of S1P_1_ and S1P_3_. These observations raised an intriguing possibility that S1P_2_ may have a distinct role in atherosclerosis from S1P_1_ and S1P_3_.

We have studied the role of S1P_2_ in atherosclerosis by using S1P_2_-deleted and non-deleted apoE^−/−^ mice [22]. The *en face* plaque area in spread aortae was dramatically reduced (approximately70%) in homozygous knockout (S1P_2_^−/−^) mice compared with S1P_2_^+/+^ mice after 16 weeks of HCD. The plaque area in heterozygous knockout (S1P_2_^+/−^) mice was intermediate between S1P_2_^−/−^ and S1P_2_^+/+^ mice, indicating that S1P_2_ has a gene dose-dependent proatherogenic effect. In the plaques of S1P_2_^−/−^ mice, the macrophage density was decreased compared with S1P2^+/+^ mice whereas SMC density in the plaques was increased in S1P_2_^−/−^ mice. Consistent with our data, Hla and colleagues very recently showed that S1P_2_-deficiency markedly inhibited atherosclerosis in apoE^−/−^ mice [23]. The mRNA expression levels of the proinflammatory cytokines TNF-α, IL-6, IFN-γ and MCP-1, and the adhesion molecule VCAM-1 were reduced in the aortae of HCD-fed S1P2^−/−^ mice compared with S1P2^+/+^ mice, whereas the phosphorylation of eNOS was increased in the aorta of S1P2^−/−^ mice [22]. The mRNA expression of S1P_1_, S1P_3_, and the S1P synthesizing and degradation enzymes including SphK-1, SphK-2, SPL and SPP1 in the aorta was not different between S1P2^+/+^ and S1P_2_^−/−^ mice. Thus, the atherosclerotic lesion is reduced in S1P_2_^−/−^ mice with the diminished inflammatory activity and the increased atheroprotective NO activity.

Our previous study using β-galactosidase (LacZ)-knockin mice at the S1P_2_ locus, in which LacZ gene expression is driven by endogenous S1P_2_ promoter, showed that S1P_2_ is expressed in ECs and SMCs of normal blood vessels in a variety of organs and the bone marrow (BM) [100]. In the atherosclerotic lesion in the aortic sinus of LacZ-knockin apoE^−/−^ mice fed HCD, macrophages, ECs, and intimal and medial SMCs were found to express S1P_2_ [22]. The role of S1P_2_ in BM-derived cells for atherosclerosis was studied by analyzing BM-chimera mice [22]. The deletion of S1P_2_ in BM cells markedly reduced atherosclerotic lesions compared with control. Thus, S1P_2_ in BM-derived cells, most likely monocytes and macrophages, play the critical role in atherosclerosis. The study by Skoura *et al.* [23] supported the importance of S1P_2_ in BM-derived cells in atherosclerosis.

**Figure 2:**
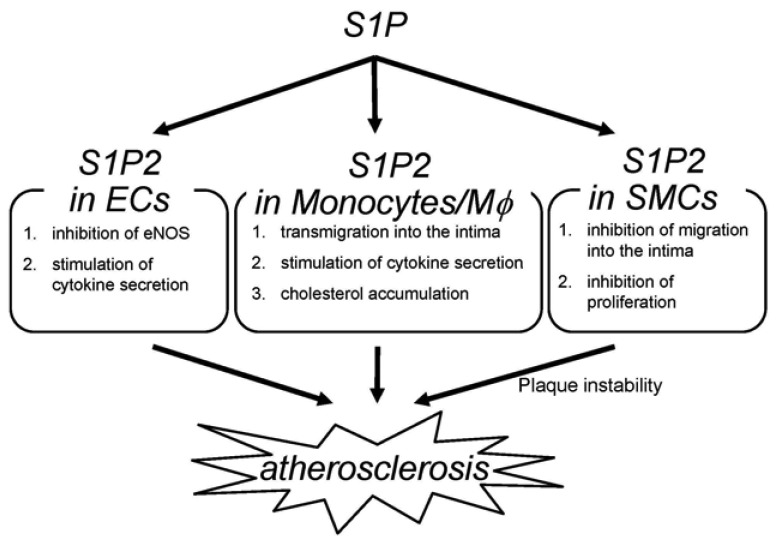
Role of S1P_2_ on atherosclerosis. S1P_2_ exerts the stimulatory effects on atherosclerosis by affecting monocytes/macrophages, ECs, and SMCs. S1P_2_ mediates inhibition of eNOS and stimulation of cytokine secretion in ECs; transmigration of monocytes, cytokine secretion and cholesterol accumulation in macrophages; inhibition of proliferation and migration into the intima of SMCs.

The deletion of S1P_2_ has a substantial impact on macrophage functions including cholesterol accumulation, cytokine production and migration (Figure 2) [22]; deletion of S1P_2_ in macrophages markedly inhibits accumulation of modified LDL through both a substantial decrease in uptake of oxidized LDLs and a modest increase in cholesterol efflux. These effects are accompanied by decreases in scavenger receptor expression (CD36 and scavenger receptor-A) and increases in cholesterol efflux transporter (ABCA1 and ABCG1) expression. These effects of S1P_2_-deficiency lead to inhibition of foam cell formation and reductions in atherosclerotic lesions. Second, deletion of S1P_2_ in macrophages inhibits the proinflammatory responses by suppressing Rho-ROCK-NF-κB signaling pathway, which is essential for the expression of the proatherogenic gene products including cytokines such as TNF-α and the scavenger receptor CD36. The ABCA1 mRNA expression is negatively regulated by ROCK through mechanisms involving LXR downregulation. Third, S1P_2_ possesses a profound influence on transmigration of monocytes/macrophages into atherosclerotic lesions. S1P_2_ mediates inhibition of macrophage migration toward a higher concentration of S1P (chemorepulsion) whereas S1P_1_ mediates stimulation of migration toward a higher concentration of S1P (chemotaxis). The blood S1P concentration is estimated to be much higher than that in tissues. The chemokines including MCP-1, which are produced in atherosclerotic lesions, attract monocytes to lesions. Therefore, S1P_2_ in monocytes very likely promotes macrophage transmigration into lesions. In fact, intravenously infused S1P_2_^+/+^ macrophages more robustly transmigrated into the vascular wall compared with S1P_2_^−/−^ macrophages. The number of total monocytes and activated monocytes (CD11b^+^Ly6C^hi^) in the peripheral blood did not differ between S1P_2_^+/+^ and S1P_2_^−/−^ mice, suggesting that S1P_2_-deficiency did not affect mobilization of monocytes to peripheral blood or their activation. These stimulatory effects of S1P_2_ on modified LDL accumulation, cytokine production, and transendothelial migration underlie the proatherogenic action of macrophage S1P_2_. Besides monocytes/macrophages, bone marrow-derived mast cells express S1P_2_, which stimulates degranulation [42]. Mast cells are implicated in plaque progression and destabilization, and therefore may contribute to the proatherogenic effect of S1P_2_ [103]. In our study, activated mast cells were reduced in the aortic wall of S1P_2_^−/−^ mice. The role of mast cell S1P_2_ in atherogenesis remains to be clarified.

S1P_2_^−/−^ ECs display altered phenotypes compared with wild-type ECs (Figure 2) [22,100]. eNOS and its product NO have atheroprotective properties. Consistent with the observation that eNOS phosphorylation is increased in the aortae of S1P_2_^−/−^ mice compared with S1P_2_^+/+^ mice, S1P_2_^−/−^ ECs show stimulation of eNOS phosphorylation in response to S1P stimulation whereas S1P_2_^+/+^ ECs exhibits a decrease in eNOS phosphorylation in response to S1P. In S1P_2_^−/−^ ECs, S1P_3_ and probably S1P_1_ mediates eNOS phosphorylation most likely through stimulation of the well known eNOS activating protein kinase Akt. In contrast, S1P induces inhibition of Akt in S1P_2_^+/+^ ECs through S1P_2_-mediated, ROCK-dependent PTEN stimulation [55], which results in inhibition of Akt and consequently eNOS [22]. S1P_2_^−/−^ ECs also showed suppression of the expression of the proinflammatory cytokines including MCP-1 and GM-CSF [22], as in the aortae [104,105]. Because both MCP-1 and GM-CSF, powerful chemoattractants for monocytes, are the NF-κB target genes, the inhibited cytokine response to S1P in S1P_2_^−/−^ ECs is due to diminished ROCK-dependent NF-κB activation. Thus, S1P_2_ in ECs could participate in atherosclerosis by regulating adhesion molecule expression, cytokine production and consequently monocyte/macrophage flux, platelet activation and thrombus formation, and intimal cell proliferation through Rho-ROCK-PTEN-mediated Akt-eNOS regulation and Rho-ROCK-NF-κB-mediated regulation of proinflammatory gene expression.

S1P_2_ deletion induces alterations of the phenotypes in SMCs (Figure 2) [22]; S1P_2_^−/−^ SMCs show enhanced proliferation in the presence of serum. This could be mediated probably at least partially by Akt stimulation due to loss of ROCK-mediated PTEN stimulation in S1P_2_^−/−^ SMCs. S1P_2_^−/−^ SMCs also exhibit loss of chemorepulsion to S1P. These phenotypes of S1P_2_-deficient SMCs might bring about a higher SMC density in atherosclerotic lesions of S1P_2_^−/−^ mice.

The lesions in S1P_2_^−/−^ mice may be stabilized compared with S1P_2_^+/+^ mice because of a lower macrophage density and higher SMC density in lesions of S1P_2_^−/−^ mice. S1P_2_^−/−^ macrophages display resistance to apoptosis induced by TNF-α and cycloheximide. Stimulated survival of S1P_2_^−/−^ macrophages may also favor plaque stabilization although the relationship between macrophage apoptosis and atherogenesis is also complex [106].

These observations in S1P_2_-deleted mice raised the intriguing possibility that pharmacological S1P_2_ blockade could afford therapeutic efficacy for atherosclerosis. JTE-K1 is a selective S1P_2_ antagonist [107]. We tested the effect of the systemic administration of JTE-K1 into HCD-fed S1P_2_^+/+^apoE^−/−^ mice for eight weeks. Oral administration of JTE-K1 (12.5 mg/kg twice daily) by gavage reduced the en face plaque area approximately by 60% with the lower density of macrophages and higher density of SMCs in atherosclerotic lesions compared with the vehicle control, thus recapitulating the phenotypes of S1P_2_^−/−^ mice [22]. The treatment of isolated macrophages with JTE-K1 suppressed uptake of DiI-acLDL and stimulated cholesterol efflux, confirming the effectiveness of S1P_2_ blockade at the cellular level. The administration of JTE-K1 did not affect food intake or body weight gain in mice over eight weeks. Mice receiving JTE-K1 did not exhibit ataxia or tilting of the trunk due to vestibular dysfunction that had been reported in S1P_2_^−/−^ mice, or any other discernible abnormality.

## 5. Concluding Remarks

Current therapy for human atherosclerotic lesions focuses on reducing the concentration of plasma LDL-associated cholesterol in the blood mainly by administering HMG CoA reductase inhibitors and other drugs [108-110]. Lowering blood cholesterol concentration leads to inhibition of the accumulation of modified LDL in the subendothelial layer, and consequently inhibition of atherosclerotic lesion formation. Although statins also exert plaque stabilizing and anti-inflammatory effects [99,108-110], no therapy to directly target foam cell formation in the face of elevated circulating LDL is currently available.

In genetic mice models for atherosclerosis, pharmacological blockade of chemoattractant receptors that are expressed in monocytes, including leukotriene B4 receptor and RANTES receptors, effectively inhibited plaque formation [111,112]. However, pharmacological blockage of leukocytes recruitment to inflammatory sites may be associated with the side effect of diminished defence mechanisms against infectious pathogens. HDL-associated S1P and the sphingosine mimetic FTY720 seem to exert atheroprotective actions via S1P_1_ and S1P_3_. In the case of FTY720, it might be necessary to separate the favorable EC-protective effect from the immunosuppressive effect.

In contrast to S1P_1_ and S1P_3_, S1P_2_ is a clearly proatherogenic receptor to act on the three cell types, macrophages, ECs and SMCs [22]. The activity of S1P_2_ seems to be diverse compared with chemokine receptors; S1P_2_ is involved in modified LDL uptake, cytokine production, migration, eNOS activation, and apoptosis in these cells. Systemic long-term administration of a selective S1P_2_-blocker can recapitulate the favorable effects of S1P_2_-deficiency without overt toxicity. As human monocytes and macrophages express S1P_2_ as in mice [83], S1P_2_ likely plays a similar role in human atherosclerotic lesion formation. Therefore, a selective S1P_2_-blocker could have clinical benefit as a new therapeutics for atherosclerosis. The combination of an S1P_2_-blocker and statins may be of great use. Thus, S1P receptors, particularly S1P_2_, are promising therapeutic targets for atherosclerosis.
